# Data-Driven Classification of Solubility Space in Deep Eutectic Solvents: Deciphering Driving Forces Using PCA and K-Means Clustering

**DOI:** 10.3390/molecules30234563

**Published:** 2025-11-26

**Authors:** Piotr Cysewski, Maciej Przybyłek, Tomasz Jeliński

**Affiliations:** Department of Physical Chemistry, Faculty of Pharmacy, Collegium Medicum in Bydgoszcz, Nicolaus Copernicus University in Toruń, Kurpińskiego 5, 85-950 Bydgoszcz, Poland; piotr.cysewski@cm.umk.pl (P.C.); m.przybylek@cm.umk.pl (M.P.)

**Keywords:** deep eutectic solvents, solubility prediction, principal component analysis, K-means clustering, pharmaceutical formulation, data-driven design, COSMO-RS

## Abstract

This study presents a robust, data-driven framework for classifying and predicting drug solubility in deep eutectic solvents (DESs), moving beyond empirical approaches to enable rational formulation design. By analyzing 2010 solubility measurements of 21 diverse pharmaceutical compounds across numerous choline chloride, betaine, and menthol-based DESs, we employed Principal Component Analysis to reduce 16 COSMO-RS-derived descriptors into four chemically interpretable dimensions explaining 86.7% of the total variance. Persistence analysis confirmed component stability, revealing two key factors: PC1 (global solvation propensity, i.e., the overall capacity of the solvent to stabilize solutes through all interaction types) and PC2 (specific interaction complementarity, i.e., the degree of matching between solute and solvent hydrogen-bonding/polarity features). K-means clustering identified four distinct solubility regimes: high-solubility DES-optimized systems (Cluster 1), reliable moderate performers (Cluster 0), intermediate candidates for optimization (Cluster 3), and fundamentally challenging combinations (Cluster 2). Comparative analysis demonstrated choline chloride’s broad utility while revealing specialized roles for menthol and betaine in specific chemical spaces. Case studies of Sulfasalazine and Caffeine illustrated how multi-cluster distributions guide formulation strategies, distinguishing precision-requiring from forgiving compounds. This taxonomy provides formulation scientists with a rational framework for DES selection, emphasizing aqueous modification, HBD and HBA diversity, and balanced solvation-interaction optimization. The integrated PCA-clustering approach transforms DES development from trial-and-error screening to targeted design, offering fundamental insights into solubility mechanisms while accelerating sustainable pharmaceutical formulation.

## 1. Introduction

Low aqueous solubility is a fundamental constraint on achieving adequate oral bioavailability [1], dose-proportional pharmacokinetics [2], and reliable, scalable manufacturability [3]. Its impact is especially pronounced for compounds in the Biopharmaceutics Classification System (BCS) Classes II and IV, where limited solubility and/or permeability governs absorption and complicates development [4]. Consequently, numerous strategies have been developed to enhance the dissolution capacity of active substances and, thereby, improve bioavailability. Representative options include salt selection to modify ionization and dissolution [5,6,7], pharmaceutical cocrystals to tune lattice energy and solute–solvent interactions [8,9], cyclodextrin inclusion complexes to raise apparent solubility and maintain supersaturation [10,11], amorphous solid dispersions to generate and sustain supersaturation during transit [12], drug nanocrystals to accelerate dissolution via increased surface area [13], lipid-based systems [14,15], and solvent or surfactant strategies including cosolvency, micellization, and pH modulation [16]. Liquid dosage forms are particularly relevant in this context: by delivering the drug already dissolved, they alleviate dissolution-rate limitations and can yield more consistent exposure than solid oral products [4]. Mechanistically, the upper bound of absorbable concentration in the intestinal lumen is set by solution thermodynamics, including nonideality and specific solubilization in bile salt–phospholipid colloids that depend on medium composition [17]. In parallel, the solid-state form and polymorphic landscape, together with nucleation and crystal-growth kinetics, govern precipitation risk and the maintenance of supersaturation during gastrointestinal transit [18].

Deep eutectic solvents (DESs) have garnered significant attention as a class of compositionally defined, tunable media engineered by selecting specific hydrogen-bond acceptors (HBAs) and donors (HBDs). Key advantages of DESs include straightforward preparation, low cost, potential biocompatibility, and high solvation power across diverse chemical compounds, positioning them as promising alternatives or complements to conventional organic solvents and ionic liquids in pharmaceutical applications [19,20]. The subfamily of natural deep eutectic solvents (NADESs) expands the palette toward bio-derived components that may offer additional processing and sustainability benefits [21,22]. NADESs represent a subclass of DESs composed of naturally occurring, often bio-derived components such as amino acids, organic acids, sugars, or choline derivatives. Typical examples include choline chloride:glycerol, choline chloride:glucose, choline chloride:xylitol, choline chloride:citric acid, and betaine:glycerol mixtures, which are entirely composed of renewable, biodegradable constituents [21,22,23,24]. Such systems have been widely employed in pharmaceutical, nutraceutical, and extraction processes owing to their low toxicity and biocompatibility [25,26]. Importantly, the ability to tailor NADES composition by combining different hydrogen bond donors (HBDs) and acceptors (HBAs) allows fine-tuning of polarity, viscosity, and hydrogen-bonding capacity, thus making them promising green alternatives to conventional solvents [27,28].

In practice, DESs are categorized based on miscibility: hydrophilic (water-miscible), hydrophobic (water-immiscible), and non-ionic systems. The resulting solubilization capacity is a multivariate function governed by the hydrogen-bond donor/acceptor balance (HBD/HBA), polarity, acidity/basicity, nanoscale segregation of polar and apolar domains, and water activity [20,29,30,31,32]. The utility of these solvents is broad: hydrophobic members, particularly menthol-based DESs, enable low-energy, selective extraction of various phytochemicals, often benefiting from ultrasound or microwave assistance for increased efficiency [22,33,34]. Conversely, hydrophilic DESs are known to markedly enhance the solubility of poorly soluble active pharmaceutical ingredients (APIs), exemplified by ibuprofen in choline chloride:xylitol:water systems [35]. They can also facilitate epithelial permeation in vitro, as shown with daptomycin across Caco-2 monolayers [36], and support efficient ultrasound-assisted extraction of polar phenolics [37].

Solubility is a pivotal physicochemical property in DES-based pharmaceutical development; measuring API solubility under controlled hydration informs rational solvent design, dissolution enhancement, and dosage-form feasibility and enables predictive structure–property modeling [31,38]. The challenge is intrinsically combinatorial because solubility depends on the identity and ratio of the hydrogen-bond acceptor and donor, temperature, and especially water content, which reorganizes DES structure and properties [39,40].

Predicting drug solubility in complex solvent systems remains a major challenge in formulation science. Traditional empirical approaches, such as Hansen solubility parameters, Hildebrand polarity indices, and trial-and-error screening, often rely on single-parameter correlations and lack transferability across structurally diverse compounds [41,42,43]. In contrast, physicochemical modeling methods, including COSMO-RS, provide a more fundamental basis by estimating activity coefficients and solvation free energies from quantum-chemical surface interactions [44,45,46]. More recently, data-driven and hybrid machine-learning models have emerged, combining quantum-chemical descriptors, molecular fingerprints, and experimental data to predict solubility across wide chemical spaces [47,48,49,50]. These strategies, ranging from multivariate regression to neural networks and graph-based representations, have shown improved accuracy and generalizability but often sacrifice interpretability.

Therefore, integrating the physically grounded insight of COSMO-RS with the pattern-recognition ability of statistical or ML techniques offers a promising route toward interpretable, predictive models of solubility in deep eutectic systems.

A practical route is to represent solubility in a low-dimensional chemical space defined by solute features, solvent descriptors, and controlled experimental factors. COSMO-RS links quantum-chemical surface charge densities (σ-profiles) to activity coefficients and solvation free energies, which supports mechanistic screening of complex media [51,52]. In parallel, data-driven models, including descriptor-based regressors and graph neural networks, use growing solubility datasets across solvents to make reliable predictions [53,54,55,56]. Incorporating physically grounded features from COSMO-RS, such as σ-moments or estimated activity coefficients, into these models often improves accuracy for solvent and formulation selection [50,57,58]. Nevertheless, interpretability and a clearly defined domain of applicability remain essential for decision-making [59]. Dimensionality reduction of COSMO-derived descriptors (σ-profiles or σ-moments) with Principal Component Analysis yields axes that separate global polarity and dispersion from specific hydrogen-bonding and acid–base complementarity; unsupervised clustering in this embedded space can reveal recurring solvent–solute regimes within deep eutectic solvent families and across API classes [60,61,62].

The aim of this study is to establish a general, data-driven framework for classifying and predicting API solubility in deep eutectic solvents, moving beyond empirical approaches to enable rational formulation design. Solute–solvent interactions are summarized using COSMO-RS descriptors and projected by Principal Component Analysis (PCA) to obtain a compact, interpretable map. K-means clustering delineates recurrent solubility regimes and the boundaries between them. Traditional approaches for predicting solubility in complex solvents are primarily empirical and rely on macroscopic correlations between experimental solubility data and a limited number of solvent parameters. The most common examples include: (i) Hildebrand solubility parameter, which estimates solubility based on cohesive energy density but assumes uniform, nonpolar systems and neglects specific interactions [63]; (ii) Hansen solubility parameters, which extend the Hildebrand model by distinguishing dispersion, polar, and hydrogen-bonding contributions, yet still treat solvent effects additively and cannot represent cooperative behavior in hydrogen-bonded networks [41]; (iii) logP-based or Abraham solvation models, correlating solubility with overall polarity or hydrophobicity, which perform well for molecular solvents but fail for multi-component, highly associated DES mixtures [64]. In contrast, the present PCA–clustering framework integrates 16 COSMO-RS-derived descriptors that directly quantify interaction energies (electrostatic misfit, hydrogen bonding, van der Waals, and chemical potential). This enables simultaneous consideration of solute–solvent complementarity, network flexibility, and crystalline stability phenomena that are inaccessible to empirical correlations. Therefore, while empirical parameters describe solubility through averaged macroscopic behavior, our data-driven approach captures molecular-scale physics underlying solvation. The PCA components represent orthogonal axes of global solvation propensity, interaction complementarity, and bulk-medium efficiency, offering interpretability comparable to empirical models but with far greater generality. Consequently, this method goes beyond empirical approaches by unifying mechanistic understanding with predictive capability, allowing rational design of DES formulations without extensive experimental screening. We compiled a curated, chemically diverse dataset spanning several HBAs (choline chloride (ChCl), betaine, and menthol) and their aqueous variants, together with various polyol HBDs, e.g., glycerol (GLY) and triethylene glycol (TEG). The analysis is designed to quantify how HBD identity and hydration shift position in this map and, in turn, the achievable mole-fraction solubility. The outcome is a set of simple, transferable rules for selecting HBAs and HBDs and steering hydration to access high-solubility regions, providing a practical starting point for DES formulation.

In summary, the approach presented here contrasts the multivariate, objective, and predictive classification framework with conventional empirical approaches, which typically rely on trial-and-error screening or single-variable correlation (e.g., relating solubility to a single bulk property like viscosity or pK_a_, neglecting the coupled nature of solvation). This framework, in contrast, leverages PCA to objectively decompose the multivariate solvation phenomenon into distinct physicochemical drivers (PC1: global solvation, PC2: specific interactions) and uses K-means to define discrete, predictive solubility regimes. This transforms DES development from screening to targeted design by providing a rational map of the chemical space, which is the core difference worth highlighting.

## 2. Results and Discussion

**Scheme 1:**
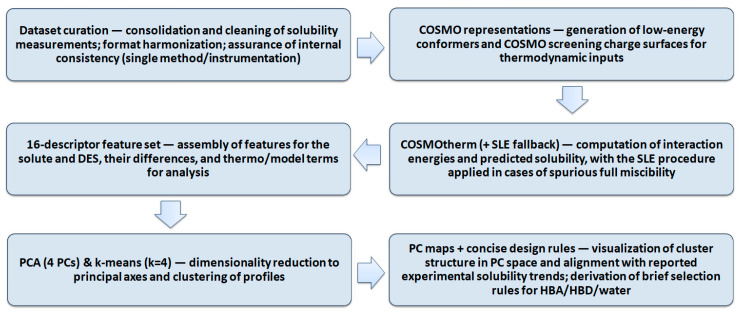
Workflow of the data-driven methodology used in this study.

To provide a clear overview of the methodological structure, the complete workflow of the study is summarized in Scheme 1. The utilized approach integrates experimental solubility data with COSMO-RS thermodynamic modeling and multivariate statistical analysis. Beginning with data curation and descriptor generation, followed by PCA dimensionality reduction and K-means clustering, each step contributes to transforming raw solubility information into interpretable physicochemical design principles. The following subsections present and discuss the obtained results in detail.

### 2.1. Principal Component Analysis

The studied dataset included 2010 solubility measurements performed for 21 different APIs in various DES systems comprising different HBAs and HBDs. In order to describe these data, a comprehensive set of 16 descriptors derived from COSMO-RS calculations was used. The explanation of the used descriptors can be found in Section 3 in Table 1. Principal Component Analysis (PCA) was used to condense these 16 COSMO-RS–derived descriptors into a smaller number of independent factors that summarize the main physicochemical influences on solubility. In simple terms, PCA transforms correlated variables into new, orthogonal axes, called principal components (PC), that capture most of the variation in the dataset. This allows complex molecular descriptors to be visualized and interpreted in a few dimensions that reflect the dominant chemical effects.

#### 2.1.1. Variance Explanation and Component Stability

**Figure 1:**
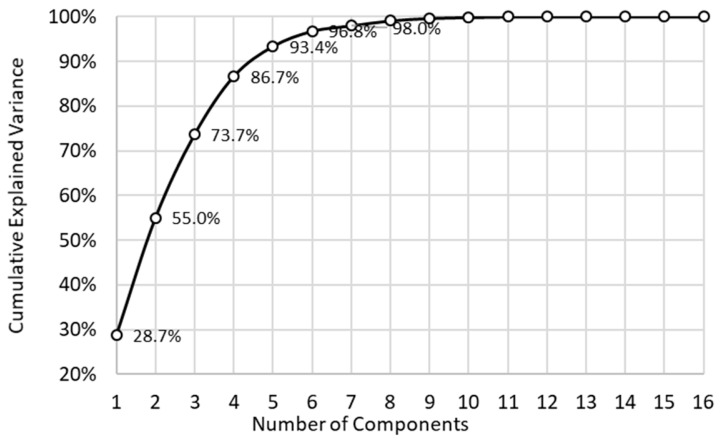
Cumulative variance explained by the number of first principal components. The first four principal components capture 86.7% of the total variance (PC1: 28.7%, PC2: 26.3%, PC3: 18.7%, PC4: 13.0%), indicating effective dimensionality reduction.

The explanation of the cumulative variance is shown in Figure 1. Analysis of variance revealed that the first four principal components (PCs) explain approximately 86.7% of the total variance (PC1: 28.7%, PC2: 26.3%, PC3: 18.7%, PC4: 13.0%), which confirms the validity of reducing the data to a 4D map that describes the dominant mechanisms affecting solubility in DESs. This level of cumulative variance is high in the context of complex multicomponent systems and indicates that the key driving forces of solubility are captured by a relatively small number of orthogonal variables.

**Figure 2:**
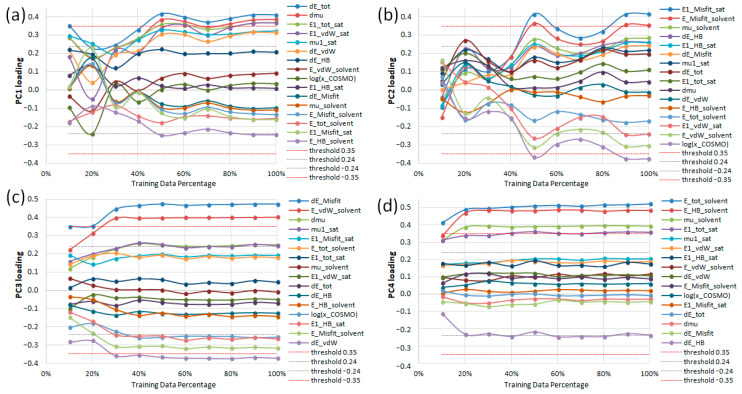
Persistence analysis of principal component loadings with significance thresholds. (**a**): PC1. (**b**): PC2. (**c**): PC3. (**d**): PC4. The curves show the stability of loading vectors as a function of training set size, illustrating how quickly each component converges toward its final form. The horizontal dashed lines mark the interpretative loading thresholds of |0.35| (strong contribution) and |0.24| (moderate contribution). The specific descriptors corresponding to these loadings for each PC are defined and detailed in Table 1.

The persistence of principal component loadings across increasing training set sizes provides crucial insights into the stability and interpretability of the derived components, as demonstrated in Figure 2. In the figure, the horizontal dashed lines at 0.35, 0.24, −0.24, and −0.35 mark thresholds of interpretative significance: loadings exceeding |0.35| indicate strong contributions of a descriptor to a given component, whereas values between |0.24| and |0.35| represent moderate but still relevant contributions. Loadings within the central range from −0.24 to +0.24 are considered negligible. Persistence analysis of the components with increasing fractions of training data revealed different rates of stabilization: PC1 stabilizes early (around 40% of the data), which suggests that global solvation factors are strong and easy to detect even with a moderate dataset size. PC2 achieves stability only with a much larger data fraction (approx. 90%), which suggests it describes subtle, specific interactions (e.g., hydrogen-bonding complementarity, local polarity matching) that require extensive sampling of the DES–solute space to become statistically invariant. This difference in stability has practical consequences for data collection strategies: quick surveys can detect general trends (PC1), but a full understanding of the role of specific interactions requires larger, more diverse measurement sets (PC2). While PC1 and PC2 capture the main variation in solubility-related features (global solvation propensity and specific interaction complementarity), the remaining components also have clear physical interpretations. PC3 describes bulk-medium efficiency, reflecting dispersion and cohesive energy effects of the DES matrix that modulate solvation beyond polarity. PC4, which contributes less than 8% of the total variance, primarily represents fine dielectric tuning and minor residual correlations among polarity-related descriptors. Although of secondary significance, PC3 and PC4 provide additional resolution within borderline systems and confirm the internal consistency of the descriptor space. Interestingly, they both stabilize quite early, i.e., around 20% of the data.

#### 2.1.2. Chemical Interpretation of Principal Components

Based on the loading vectors and correlations with the COSMO-RS energy components, we interpret the components as follows:

(a) PC1—global solvation propensity axis (Figure 2a).

PC1 represents the overall tendency of a compound to dissolve. Systems with high PC1 values are characterized by favorable total interaction and van der Waals energies, together with strong COSMO-RS–predicted solubility. Negative PC1 values, in contrast, correspond to compounds dominated by crystalline stability and electrostatic mismatch. In practical terms, PC1 measures how easily a solute can leave its crystal lattice and interact with the solvent, i.e., it is a general “solvation power” axis. Taking into account the specific descriptors (please refer to Table 1 in Section 3), the loading pattern is dominated by positive contributions from COSMO-RS predicted solubility (log(x_COSMO)), total energy differences (dE_tot), and van der Waals interactions (dE_vdW), contrasted with negative loadings for misfit energies (dE_Misfit, dE_Misfit_sat) and fusion Gibbs free energy (ΔG_fus). The strong contribution of COSMO-RS predictions validates the physical relevance of this component, while the opposition between solvation drivers and crystalline stability captures the fundamental thermodynamic balance governing dissolution. Hence, PC1 represents the scale and magnitude of non-specific stabilization. Systems positioned highly along the PC1 axis offer maximum global stabilization, independent of the solute’s specific hydrogen-bonding demands.

(b) PC2—specific interaction complementarity axis (Figure 2b).

PC2 reflects how well the solute and solvent match in terms of hydrogen bonding and polarity. High PC2 scores indicate good complementarity, particularly efficient hydrogen bonding and polarity alignment, while low PC2 values denote mismatched donor/acceptor features that limit solubility. This axis therefore distinguishes systems with strong, specific interactions from those governed mainly by bulk solvation. PC2 loadings show strong positive contributions from solvent misfit energy (E_Misfit_solvent), hydrogen bonding components (E_HB_solvent, dE_HB), and solvent chemical potential (µ_solvent). The prominence of solvent-specific descriptors in PC2 underscores the critical importance of DES composition in determining solubility behavior, reflecting the unique, tunable nature of deep eutectic solvents compared to conventional molecular solvents.

(c) PC3 and PC4—subtle energetic balances (Figure 2c,d).

The third and fourth components capture more subtle energetic balances, with PC3 emphasizing solvent bulk properties and PC4 focusing on hydrogen bonding specificity. These components, while explaining smaller fractions of variance individually, collectively contribute to capturing the nuanced interactions that differentiate various DES formulations and solute types.

#### 2.1.3. Implications for Deep Eutectic Solvent Design

The chemical interpretation of the four principal components provides actionable insights for rational formulation of DESs. Instead of treating PCA solely as a statistical reduction tool, here it is used as a conceptual framework that links molecular descriptors to design strategies. Each principal component highlights a different “design lever” that can be intentionally manipulated when formulating eutectic systems for poorly soluble pharmaceuticals.

PC1 functions as a universal metric for identifying promising solvent systems. Formulations with high PC1 values are expected to provide adequate solvation regardless of detailed composition. For formulation scientists, this means PC1 can serve as an initial filter: compounds mapping to high-PC1 regions are strong candidates for successful DES solubilization, whereas those in low-PC1 regions may require structural modification of the solute or unconventional solvent choices.

PC2 captures hydrogen bonding efficiency, polarity matching, and misfit correction between solute and solvent. It highlights that even if a DES exhibits strong global solvation capacity (high PC1), poor matching of functional groups may drastically limit solubility. Conversely, moderate-PC1 systems can still achieve high solubility if PC2 is optimized. This axis therefore points directly to the importance of hydrogen bonding properties necessary for fine-tuning.

The persistence analysis shows that while PC1 stabilizes with relatively small datasets, PC2 requires extensive sampling to reliably capture interaction-specific effects. This finding has two important implications. Firstly, smaller datasets are sufficient to detect global solubility trends, but more comprehensive studies are required to uncover subtle, interaction-driven solubility enhancements. Secondly, machine learning or regression models trained on limited data may reproduce PC1-like behavior (general solvation trends) but will fail to capture PC2 unless enriched datasets are used. Thus, rational DES design requires intentional sampling across diverse solute classes to ensure both global and specific contributions are represented.

To illustrate the practical implications of the PCA results, several representative cases can be considered. For instance, syringic acid and caffeic acid, which both exhibit high PC1 values, are located in regions of the score plot associated with strong solvation propensity and efficient hydrogen-bonding interactions. These compounds were experimentally found to dissolve well in choline chloride:glycerol and choline chloride:ethylene glycol systems, in agreement with their predicted position in the high-PC1/high-PC2 quadrant. In contrast, p-coumaric acid displays a lower PC1 score, indicating a stronger influence of crystal stabilization and limited compatibility with typical hydrogen-bonding DESs. This trend suggests that such compounds would require either more polar NADESs (e.g., choline chloride:citric acid) or mixed systems with higher water content to enhance solubility. These examples show how the PCA map can serve as a practical guide for formulation design: solutes positioned at high PC1 values are likely to benefit from classical hydrogen-bonding DESs, whereas those at low PC1/high PC2 regions may require more tailored solvent environments or co-solvent adjustments.

Overall, the robust 86.7% variance captured with only four components suggests that despite the apparent complexity of DES systems, the fundamental solubility drivers are comprehensible and can guide rational solvent design. This significant dimensional reduction proves that the fundamental factors driving solubility are comprehensible, paving the way for rational solvent design. The methodology used, which integrated persistence analysis with the chemical interpretation of the components, offers a powerful and insightful template for studying other complex solvent systems. This approach provides both a deeper fundamental understanding and highly practical tools for solvent selection and design in the pharmaceutical field.

### 2.2. Identification of Distinct Solubility Regimes Using K-Means Clustering

**Figure 3:**
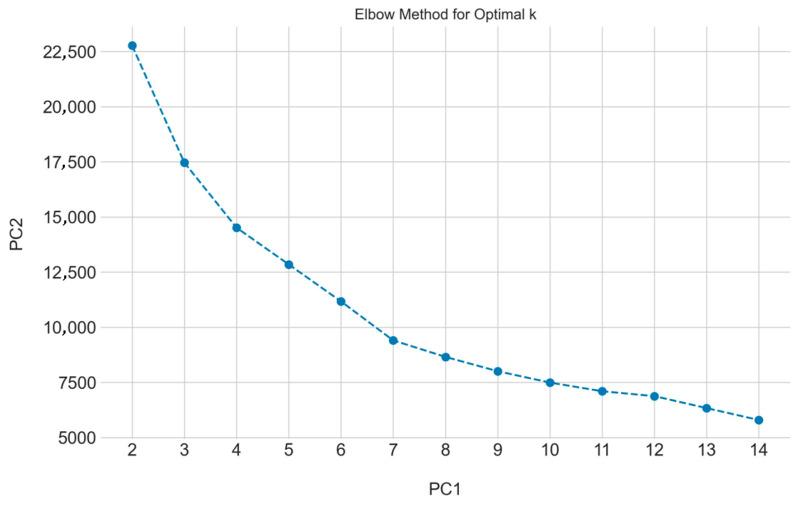
Elbow method analysis for determining the optimal number of clusters (k) in the PCA-reduced solubility space. The *Y*-axis represents the within-cluster sum of squares (inertia). The pronounced elbow visible at k = 4 indicates the optimal balance between cluster cohesion and model complexity.

Following dimensionality reduction by PCA, the next step was to partition the chemical space into coherent regions that reflect distinct solubility behaviors. To this end, K-means clustering was applied, and the optimal number of clusters was determined using the elbow method (Figure 3).

K-means clustering grouped the DES–solute systems into categories with similar solvation behavior, based on their positions in the PCA space. In practical terms, the algorithm assigns each system to one of k groups by minimizing the distance between its PCA coordinates and the average position (centroid) of that group. The inertia values decreased sharply from ∼22,500 at k = 2 to ∼17,500 at k = 4, after which the slope of the curve flattened considerably. This pronounced “elbow” at k = 4 indicates that four clusters achieve the optimal balance between explanatory power and parsimony: the partition captures the dominant patterns of solubility variation while avoiding the pitfalls of overfitting associated with excessive cluster numbers. Importantly, the choice of k = 4 is not only statistically justified but also chemically meaningful. It resonates with the PCA results, where four principal components together explained 86.7% of the variance. Thus, the four-cluster solution provides a natural extension of the dimensionality reduction: each cluster corresponds to a specific combination of solvation-related descriptors encoded in PC1–PC4. This coherence ensures that clusters are not arbitrary statistical constructs but instead reflect real physicochemical regimes governing solubility in DESs.

From a methodological perspective, the adoption of four clusters also guarantees that each group is sufficiently populated for robust statistical interpretation. Unlike higher-k solutions (k = 5 or k = 6), which continue to reduce inertia marginally but fragment the dataset into smaller groups with limited generalizability, the four-cluster solution maintains both granularity and interpretability. The diminishing returns beyond k = 4 underscore that additional clusters would capture noise or local fluctuations rather than fundamental solubility mechanisms.

### 2.3. Chemical Space Partitioning with Clusters

**Figure 4:**
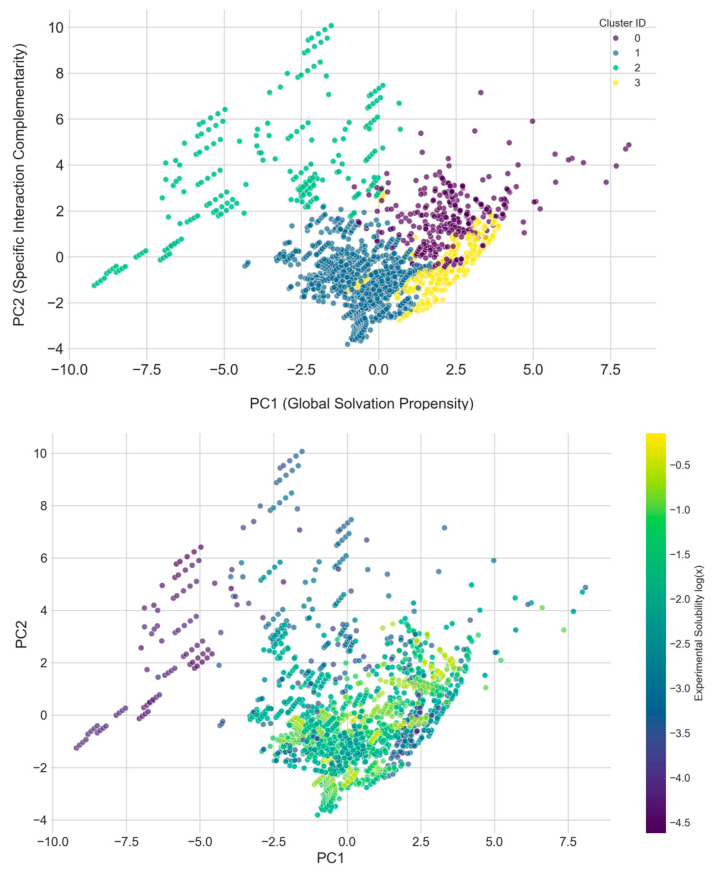
Map of the deep eutectic solvent (DES) solubility space. (**Top panel**): The location of all DES–solute data points in the space defined by PC1 (global solvation propensity) and PC2 (specific interaction complementarity), clustered using K-means (k = 4). (**Bottom panel**): The same space, where each data point is colored according to its experimental solubility logarithm value. The alignment of the clusters with the solubility gradient demonstrates that PC1 and PC2 effectively isolate the fundamental mechanisms governing solubility.

K-means clustering was applied to the four-dimensional chemical space defined by the principal components (PC1–PC4) to partition the solubility data into distinct, chemically meaningful regimes. The optimal number of clusters, k = 4, was determined using the elbow method (Figure 3). This clustering successfully separates the solute and deep eutectic solvent combinations, allowing for a comprehensive visualization and interpretation of the solubility landscape, particularly in the PC1–PC2 projection (Figure 4).

#### 2.3.1. Cluster Separation and Solubility Space Mapping

The application of K-means clustering with the optimal number of clusters, k = 4 (determined by the elbow method), resulted in an effective and chemically meaningful segmentation of the solubility chemical space. Each of the four identified clusters occupies a unique and distinctly separated region on the plane defined by PC1 and PC2 (Figure 4, top panel). This clear spatial separation confirms that the clusters represent fundamentally different solubility regimes in DES–solute systems, driven by distinct combinations of the underlying physicochemical factors. Cluster 0 (violet color in the top panel of Figure 4) primarily occupies the high-PC1 and moderate-PC2 region. Cluster 1 (blue) is concentrated in the moderate-PC1 and high-PC2 quadrant. Cluster 2 (green) dominates the low-PC1 and moderate-PC2 region. Cluster 3 (yellow) is distributed across moderate-PC1 values with variable PC2. The clustering resulting in four non-overlapping regimes, each occupying a distinct region of the principal component space, indicates that each cluster represents a fundamentally different combination of the underlying physicochemical properties captured by PC1 (global solvation propensity) and PC2 (specific interaction complementarity).

The chemical significance of this partitioning was definitively validated by visually superimposing the experimental solubility logarithm onto the reduced space. This visualization reveals a striking solubility gradient that is strongly correlated with the cluster position, allowing for the designation of two specific and distinct solubility regions. The high solubility region (yellow/green colors in the bottom panel of Figure 4) is concentrated primarily within Cluster 1 and portions of Cluster 3. It corresponds to the optimal balance between favorable global solvation (PC1) and good specific interaction matching (PC2), exhibiting log(x) values approaching −1.0 to −0.8. The low solubility region (blue/purple colors) is dominated by Cluster 0 and Cluster 2. Low solubility is associated with either poor global solvation (low PC1) or suboptimal specific interactions (extreme PC2 values). This region exhibits log(x) values typically ranging from −4.0 to −2.4.

#### 2.3.2. Interpretation of Cluster Characteristics

Based on their positioning in the chemical space and their associated solubility patterns, the four clusters can be chemically interpreted as distinct solubility regimes:

(a) Cluster 0 (violet)—Challenging Solutes with High Crystalline Stability.

**Figure 5:**
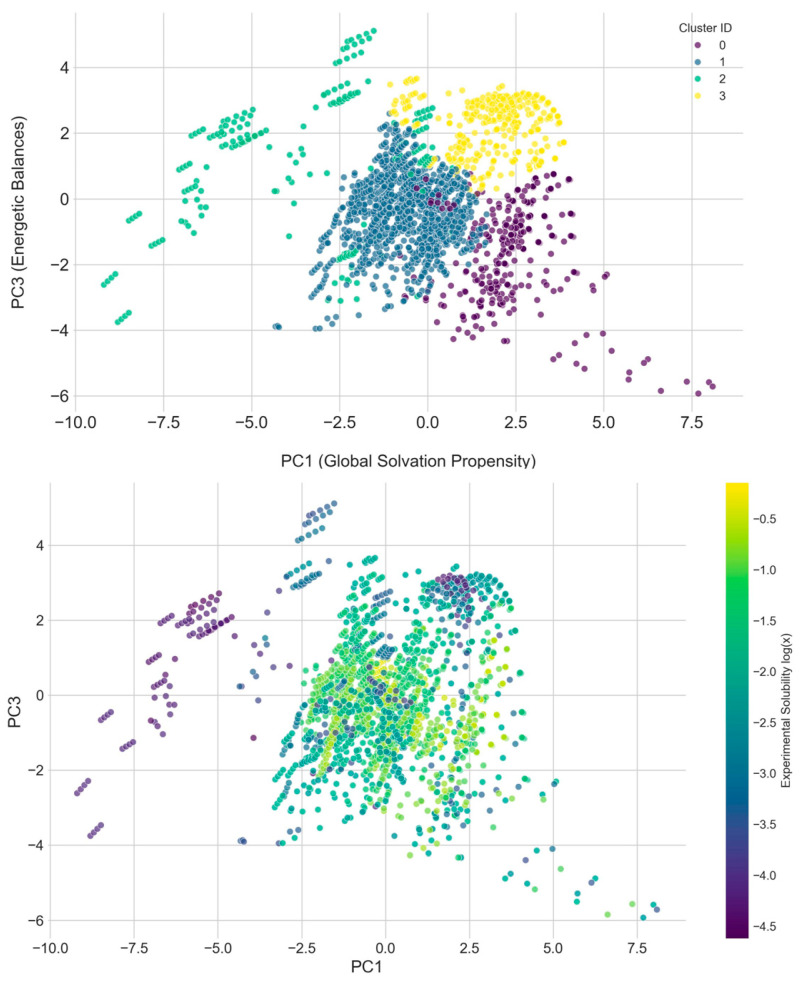
Map of the deep eutectic solvent (DES) solubility space. (**Top panel**): The location of all DES–solute data points in the space defined by PC1 (global solvation propensity) and PC3 (solvent bulk properties), clustered using K-means (k = 4). (**Bottom panel**): The same space, where each data point is colored according to its experimental solubility logarithm value.

Located in the high PC1, moderate PC2 region (Figure 4), this regime is associated with compounds whose inherent solubility limitations (high crystalline stability) override the DES’s reasonable global solvation potential. The PC1–PC3 projection (Figure 5) reveals this cluster is tightly constrained to low PC3 values, indicating a limitation related to unfavorable bulk solvent properties, such as high viscosity or cohesive energy density. Solubility is generally low to moderate (log(x) ≈ −3.2 to −2.4), as confirmed by the blue/purple coloring in the figure. This combination of high PC1 but low PC3 and solubility suggests that for these solutes, even a DES with good overall interaction energy cannot overcome the combined barriers of a rigid solute lattice and a DES with inefficient bulk properties.

(b) Cluster 1 (blue)—DES-Optimized Solutes.

Characterized by moderate PC1 and high PC2 (Figure 4), this cluster represents the optimal balance of properties. The solutes exhibit excellent specific interaction complementarity (high PC2), particularly hydrogen bonding, with the DES components. This is the primary driver for their high solubility (log(x) ≈ −1.0 to −0.8, yellow/green in Figure 4). In the PC1–PC3 space (Figure 5), Cluster 1 shows a broad distribution, achieving high solubility across a wide range of bulk property efficiencies (PC3). This demonstrates that while global solvation (PC1) must be adequate, the precise matching of specific interactions (PC2) is the dominant factor for achieving peak performance, and this can be realized through multiple combinations of bulk solvent characteristics.

(c) Cluster 2 (green)—Global Limited Solvation.

Positioned at low PC1 with variable PC2 (Figure 4), solubility in this cluster is fundamentally limited by poor overall solvation energetics in the DES environment. This results in the lowest observed solubility (log(x) ≈ −4.0 to −2.6, dark blue in Figure 4). The PC1–PC3 view (Figure 5) shows these systems span a wide range of PC3 values, indicating that the solvation failure is rooted in the global descriptor (PC1) and cannot be rescued by tuning bulk properties (PC3) or even by reasonable specific complementarity (PC2). This represents a fundamental mismatch between the solute and the DES milieu.

(d) Cluster 3 (yellow)—Versatile Intermediate Solubility.

This cluster features a broad distribution across moderate PC1 values and a wide range of PC2 (Figure 4), representing a chemically diverse group with reasonable global solvation potential. Its most defining characteristic is its wide solubility range (log(x) ≈ −2.6 to −0.8), covering the spectrum from the upper edge of Cluster 2 to the lower edge of Cluster 1. In the PC1–PC3 projection (Figure 5), it also shows significant dispersion, occupying a central space between Clusters 0, 1, and 2. This intermediate and variable positioning indicates that these systems are highly sensitive to fine-tuning. Small compositional changes that adjust specific interactions (shifting PC2) or bulk efficiency (shifting PC3) can readily push a formulation from moderate into high solubility, making this cluster a prime target for systematic optimization.

#### 2.3.3. Implications for DES Selection and Design

The data-driven taxonomy established through PCA and K-means clustering provides a robust and rational framework for the selection and design of deep eutectic solvents in pharmaceutical applications, moving decisively beyond traditional trial-and-error screening methodologies. The separation of the chemical space into four distinct clusters reveals several fundamental principles for effective drug formulation.

Cluster 1 represents the ideal target space for drug formulation. This cluster, defined by an optimal balance of moderate PC1 and high PC2, signifies DES–solute combinations with maximum solubility enhancement. For drug formulation, the goal should be to strategically adjust either the solute or the DES composition to move the system into the Cluster 1 regime, indicating that the specific intermolecular interactions are optimized.

Cluster 2, although corresponding to low-solubility systems, is still informative. It delineates formulations where strong crystal stabilization or polarity mismatch dominate, helping to identify unpromising solvent combinations early. Such “negative” clusters define the boundaries of solvation performance and indicate strategies, such as increasing polarity or water fraction, to move systems toward more favorable regimes.

PC2 emerges as the critical design parameter. While global solvency (PC1) sets the baseline, the PC2 axis (specific interaction complementarity) is the key differentiating factor for achieving exceptional solubility. High PC2 values are consistently and strongly associated with enhanced solubility, confirming that the precise matching of specific forces (e.g., hydrogen bond donor/acceptor complementarity) is more crucial than bulk properties for achieving high drug loading.

The clear separation between Cluster 0 and Cluster 1 provides a direct design strategy. Cluster 0 systems are limited by the solute’s high crystalline stability, despite favorable global solvation potential. By focusing on strategies to maximize PC2 (the Cluster 1 characteristic)—such as fine-tuning the DES composition to better complement the solute’s H-bonding groups—formulators may be able to destabilize the crystal lattice and overcome these inherent solubility hurdles. This provides a mechanism for prioritizing interaction design over simple solvent strength.

The broad solubility range and intermediate positioning of Cluster 3 indicate that these systems are highly sensitive to small compositional changes. This suggests significant opportunities for further solubility enhancement through minor adjustments to the DES composition, such as controlled addition of water (aqueous modification) or slight changes in the hydrogen bond donor/acceptor ratio. The wide dispersion makes this cluster an excellent candidate for systematic, local optimization campaigns.

In essence, the strong agreement between the unsupervised clustering and the experimental solubility values demonstrates that the principal components successfully capture the essential physics governing drug dissolution in DESs. This data-driven taxonomy provides a robust, actionable framework for selecting or designing DESs for specific pharmaceutical compounds, accelerating the discovery and optimization process.

#### 2.3.4. Multi-Dimensional Cluster Characterization

Examination of the PC1–PC3 projection (Figure 5) provides crucial complementary insights that significantly enhance the chemical interpretation of the four solubility regimes, particularly by revealing patterns not fully captured in the primary PC1–PC2 plane.

While the fundamental four-regime structure remains consistent, the introduction of the third principal component (PC3: solvent bulk properties) clarifies the nuanced differences in solubility limitation mechanisms across the clusters:

(a) Cluster 0 (violet)—challenging solutes.

In the PC1–PC3 space, this cluster is compressed along the PC3 axis (low–moderate PC3) while maintaining consistently high PC1. This indicates homogeneity in terms of bulk solvent properties and hydrogen bonding, with the main limitation arising from the strong crystalline stability of the solute. For these compounds, improving bulk solvent parameters is unlikely to overcome solubility barriers; instead, strategies targeting lattice disruption (e.g., thermal modulation, additives breaking crystal packing) may be more effective.

(b) Cluster 1 (blue)—DES-optimized solutes.

This cluster shows pronounced dispersion along PC3 despite moderate PC1 and high PC2. This variability reveals internal subgroups: some systems achieve high solubility through favorable bulk properties, while others succeed due to specific hydrogen-bond complementarity. Practically, this demonstrates that high solubility is attainable through multiple design pathways.

(c) Cluster 2 (green)—global limited solvation.

Members of this cluster are characterized by low PC1 values combined with moderate–high PC3. Importantly, separation from Cluster 0 along the PC3 axis indicates a distinct limitation mechanism: while crystalline constraints dominate Cluster 0, the bottleneck here is inadequate global solvation. Despite having acceptable bulk features (PC3) and sometimes reasonable PC2 contributions, these solutes remain poorly soluble due to mismatched polarity and insufficient dielectric compatibility.

(d) Cluster 3 (yellow)—versatile intermediate solubility.

This cluster shows the broadest distribution in both PC1–PC2 and PC1–PC3 projections, confirming its heterogeneous nature. The spread along PC3 highlights the chemical diversity of this group. Cluster 3 therefore represents a versatile exploration zone, where small compositional adjustments may shift formulations toward either high or low solubility regimes.

Overlaying solubility data onto the PC1–PC3 plane further reveals systematic trends:

(a) High-solubility hotspot emerges in the moderate PC1–moderate PC3 region, populated by parts of Cluster 1 and Cluster 3. This zone represents an optimal balance between global solvation, specific interactions, and favorable bulk properties.
(b) Two low-solubility regions are clearly distinguished: (i) high PC1 with low PC3 (Cluster 0), where crystalline lattice effects dominate, and (ii) low PC1 across any PC3 (Cluster 2), where poor global solvation is the bottleneck.
(c) While PC1 largely sets the baseline solubility, PC3 fine-tunes the outcome, particularly for compounds in the intermediate PC1 range. This suggests that for borderline cases, manipulating PC3-related features may be the most effective route to improvement.

The PC1–PC3 projection confirms that while PC1 sets the baseline solubility potential, effective optimization of DES systems requires at least three dimensions: PC1 for global solvation propensity, PC2 for specific interaction complementarity, and PC3 for bulk solvent properties. By revealing subpopulations, distinct limitation mechanisms, and new optimization pathways, the PC1–PC3 analysis underscores the multifaceted nature of solubility challenges in deep eutectic solvents. It also indicates important strategies in the design of eutectic systems. For instance, instead of a linear trial-and-error approach, the PC1–PC3 map helps identify which lever, raising global solvation (PC1) versus optimizing bulk properties (PC3), is most promising for a given API. Furthermore, the dispersion along PC3 shows that even highly soluble systems may require different design routes, which justifies testing chemically diverse HBDs even when high solubility is already indicated. It is also reasonable to assume that for compounds near cluster boundaries, targeted experiments modifying PC3-related parameters are more likely to succeed than random screening.

#### 2.3.5. A Practical Workflow for Formulation Design

The primary utility of this data-driven taxonomy is to provide a rational, chemistry-informed workflow for designing DES formulations, thereby replacing traditional trial-and-error screening. The following step-by-step procedure is proposed for a new, poorly soluble API: (i) Descriptor Calculation & Positioning: Compute the 16 COSMO-RS descriptors for the API with a small set of candidate DESs (e.g., 5–10 common combinations like ChCl:GLY, ChCl:TEG, and Betaine:GLY). Project these systems into the established PCA space to determine their initial cluster membership. (ii) Diagnosis & Strategy Selection: If the system falls in Cluster 1, the formulation is near-optimal. Focus shifts to fine-tuning for secondary properties (e.g., viscosity, stability). If the system falls in Cluster 3, it has high optimization potential. The goal is to shift it into Cluster 1 by deliberately tuning the DES composition. If PC2 is low, prioritize enhancing specific interactions by switching to an HBD with better complementarity (e.g., from glycerol to TEG). If PC1 is low, focus on improving global solvation by testing more potent HBAs (e.g., from menthol to choline chloride) or adjusting water content. If the system falls in Cluster 0, the limitation is a combination of solute crystallinity and poor bulk properties. Strategies should include exploring co-solvents or additives that disrupt the crystal lattice or investigating ternary DESs with components that significantly alter the bulk efficiency (PC3). If the system falls in Cluster 2, it indicates a fundamental incompatibility. This signals that conventional DESs are unlikely to be effective, and resources should be directed towards alternative formulation technologies (e.g., amorphous solid dispersions, nanocrystals) early in the development process. (iii) Targeted Experimentation: Based on the diagnostics above, perform a focused set of experiments (e.g., testing 2–3 strategically chosen new HBDs or a water content gradient) rather than a broad, untested screen. This approach dramatically reduces the experimental time and cost required to arrive at an optimal formulation.

This framework offers a transformation of DES formulation from an empirical art into a targeted engineering process, where each experimental iteration is guided by an understanding of the underlying solubility regime.

### 2.4. DES Composition Patterns Across Solubility Regimes

The clustering analysis not only delineates distinct solubility regimes but also uncovers systematic patterns in deep eutectic solvent composition associated with each regime. Examining the distribution of HBAs, HBDs, and water content across clusters provides key insights into why some solvent systems excel while others underperform. These composition-specific patterns translate directly into practical formulation guidelines, bridging the gap between statistical clustering and chemical design. To further elucidate the physicochemical drivers behind the cluster taxonomy, a quantitative analysis of the dominant DES components and their link to the principal components was performed. This reveals that each cluster is defined not just by solubility outcome, but by a specific solvation mechanism dictated by its composition.

#### 2.4.1. Cluster Characteristics and DES Component Distribution

Each cluster is characterized by a unique profile of DES components, solute coverage, and solubility outcomes, highlighting different formulation strategies and limitations:

(a) Cluster 0—challenging solutes.

With 324 samples, Cluster 0 has a moderate size and is similarly moderately diverse. Choline chloride overwhelmingly dominates the HBA space (99.1%), with glycerol (26.5%) and glucose (13.6%) as favored HBDs. Importantly, this cluster contains exclusively aqueous DESs, reflecting a reliance on water to achieve even moderate solubility performance. The average solubility (−1.57 ± 0.86 log(x)) is rather good, but the narrow chemical coverage suggests this cluster represents focused yet limited formulation strategies.

(b) Cluster 1—DES-optimized solutes.

This cluster represents the most successful formulation space, containing the largest dataset (1201 samples). It also shows the highest compositional diversity, with three HBAs and fifteen HBDs represented. Choline chloride (64.4%) and betaine (26.9%) dominate as HBAs, while TEG (38.2%) and GLY (15.7%) emerge as leading HBDs. Nearly all formulations in this cluster contain water (96.6%), underscoring the critical role of aqueous modification. The cluster exhibits excellent mean solubility (−1.42 ± 0.66 log(x)) with the broadest solubility range (−3.81 to −0.15), indicating that DESs in this regime are robust and adaptable across diverse APIs.

(c) Cluster 2—global limited solvation.

Representing the smallest and most problematic group (214 samples), Cluster 2 corresponds to intrinsically difficult solute–DES pairs. It covers only six unique solutes, reflecting both dataset bias and fundamental chemical limitations. Choline chloride is the dominant HBA (90.7%), while glycerol is strongly favored as HBD (54.2%). Despite this focused composition, the cluster performs poorly (−3.46 ± 0.78 log(x)), with very limited solubility improvement possible. This suggests that these solutes may require unconventional strategies.

(d) Cluster 3—versatile intermediate solubility.

Containing 362 samples, this cluster includes choline chloride (77.6%) and betaine (22.4%) as dominating the HBA distribution, with glycerol (30.7%) and TEG (19.9%) serving as preferred HBDs. The resulting mean solubility (−2.15 ± 0.80 log(x)) is intermediate, suggesting these formulations are functional but not optimal. Their balanced composition points to a “middle ground”, i.e., systems with potential that require further tuning to achieve performance comparable to Cluster 1.

#### 2.4.2. DES Design Principles Emerging from Cluster Analysis

Several overarching principles emerge from comparing clusters, pointing to systematic formulation rules.

The first aspect is the selection of an appropriate HBA. Choline chloride dominates across all regimes, confirming its status as the “universal” pharmaceutical HBA. However, betaine shows a strong association with high-solubility formulations in Cluster 1, suggesting that alternative HBAs can enhance performance when paired with the right HBD. The near-exclusive reliance on choline chloride in lower-performing clusters also highlights an underexplored opportunity for diversifying HBA selection.

In terms of HBD optimization, glycerol appears ubiquitously but shows variable success, reflecting its context-dependent performance. In contrast, TEG is disproportionately enriched in Cluster 1 (38.2%), strongly suggesting its superiority as an HBD in high-solubility DESs. Importantly, Cluster 1 also has the broadest HBD diversity (21 types), reinforcing the principle that exploring multiple HBD families increases the likelihood of finding optimal matches.

The significant role of water is another key design principle. Nearly all clusters rely heavily on water-modified DESs (96–100%), underlining the importance of aqueous adjustment in pharmaceutical systems. Notably, only Cluster 1 includes a small fraction of successful dry DESs (3.4%), suggesting that while anhydrous DESs may work in select cases, aqueous modification remains indispensable for broad solubility enhancement. Numerous experimental studies confirm that controlled hydration is indeed a key factor enhancing solubility in deep eutectic solvents. Hammond et al. demonstrated a nanoscale transition from an ionic to an aqueous-like regime as water content increases, markedly improving mass transport and solute accessibility [39]. Similarly, Kivelä et al. observed that even limited hydration significantly alters the microstructure of hydrophobic DESs, generating mixed domains that promote dissolution of polar and amphiphilic molecules [40]. In the pharmaceutical context, Lomba et al. reported that hydrated choline chloride:xylitol:water systems markedly increased ibuprofen solubility [35]. Within the present PCA framework, these effects are reflected by systematic shifts along the PC3 and PC2 axes: water addition increases PC3 values through reduced viscosity and enhanced polarity, while moderate hydration also improves PC2 by strengthening donor–acceptor complementarity within the reorganized hydrogen-bond network. Thus, the experimental findings and our component-based interpretation are fully consistent: controlled aqueous modification acts as a quantitative regulator of both global solvation and specific interaction efficiency in DESs.

Finally, Cluster 1 demonstrates the highest number of samples per solute (80.1), indicating both extensive optimization efforts and robust reproducibility. Other clusters, with far fewer samples per solute, may reflect either inherent chemical limitations or simply underexplored formulation spaces.

Overall, the cluster-based composition analysis highlights that the most promising strategy for pharmaceutical applications is the use of choline chloride– or betaine-based aqueous DESs with polyol HBDs such as TEG and glycerol. High-solubility formulations (Cluster 1) combine HBD diversity with water inclusion, demonstrating that systematic exploration of donor families is a powerful tool for overcoming solubility barriers. In contrast, the persistent low performance of Cluster 2 suggests that some APIs face fundamental solubility limitations within conventional DES frameworks. For these challenging systems, alternative strategies may be required, such as mixed HBAs, ternary DES, or hybrid DES–co-solvent systems.

The cluster analysis also points toward several clear directions for accelerating rational DES development. This includes: (i) expanding the HBA palette beyond choline chloride and betaine, (ii) systematic HBD exploration, prioritizing polyols like TEG and glycerol but extending into carboxylic acids, amides, and sugar derivatives, (iii) fine-tuning water content to balance solubility improvement against stability and hygroscopicity concerns, and (iv) using targeted formulation strategies guided by cluster membership predictions.

### 2.5. Detailed Analysis of DES Components and APIs

#### 2.5.1. Hydrogen Bond Acceptors

**Figure 6:**
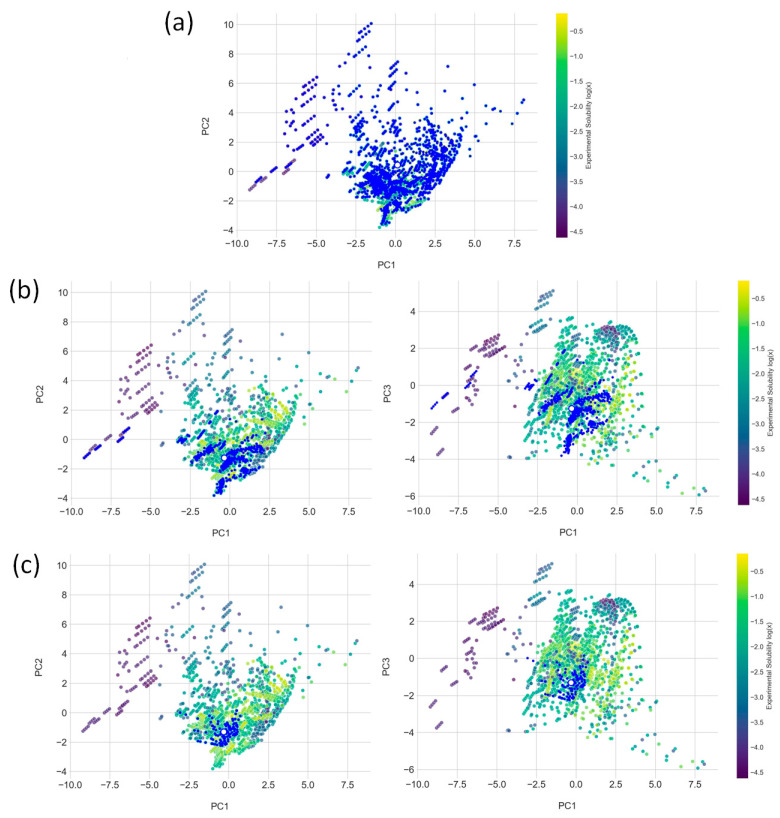
HBA-specific projections of the DES solubility space: (**a**) choline chloride (ChCl) (PC2 vs. PC1), (**b**) betaine (PC2 vs. PC1 and PC3 vs. PC1), and (**c**) menthol (PC2 vs. PC1 and PC3 vs. PC1). Points are colored by the experimental solubility, log(x)exp, from the curated dataset (references [71,72,73,74,75,76,77,78,79,80]).

Within the established PCA framework, the hydrogen bond acceptor emerges as the primary control knob that determines which neighborhoods of the solubility space become accessible. Changing the acceptor redirects formulations mainly along interaction-sensitive directions, with a secondary adjustment of bulk features, while the global placement remains set by the solute. The HBA-resolved views in Figure 6 make this effect explicit by contrasting choline chloride, betaine, and menthol.

In the choline chloride (ChCl) panel (PC2 vs. PC1), the points span the widest area, marking this HBA as the reference landscape across solubility regimes. Because its PC1–PC3 distribution closely mirrors the global map, only the PC2 vs. PC1 projection is displayed for choline chloride in the HBA-resolved view. This role of choline chloride as a convenient baseline is consistent with its early and continued prominence in DES research [19,65].

Betaine populates regions that complement choline chloride. In the PC2 vs. PC1 view, it concentrates within the central band associated with favorable solubility, while in the PC3 vs. PC1 view, it lies along a moderate-PC3 corridor that coincides with high-solubility neighborhoods on the global map. These patterns are aligned with independent reports that betaine-based DESs exhibit strong donor–acceptor matching and distinctive thermophysical behavior relative to choline systems [66,67,68].

Menthol occupies a more compact locus in the HBA-filtered maps, consistent with a specialized role. Successful formulations cluster within narrow PC windows, which matches literature describing menthol-based hydrophobic DESs that create nonpolar microenvironments with performance that is sensitive to composition and water content [40,69,70]. Taken together, HBA choice should be treated as coupled to HBD identity and water addition, since these levers jointly tune PC2 and PC3 and thereby modulate access to high-solubility regions.

#### 2.5.2. Hydrogen Bond Donors

**Figure 7:**
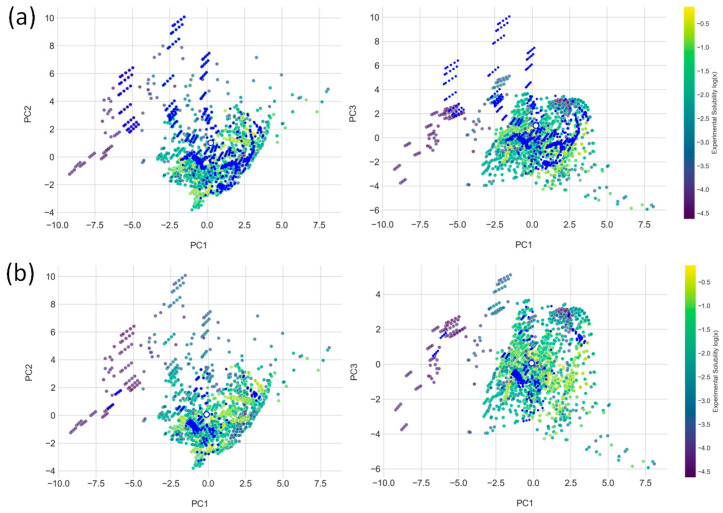
HBD-resolved projections of the DES solubility space. Formulations containing (**a**) glycerol (GLY) and (**b**) triethylene glycol (TEG) are shown as PC2 vs. PC1 and PC3 vs. PC1. Points are colored by the experimental solubility, log(x)exp, from the curated dataset (references [71,72,73,74,75,76,77,78,79,80]).

Figure 7 presents glycerol (GLY) and triethylene glycol (TEG) as PC2 vs. PC1 and PC3 vs. PC1 projections. Points are colored by the values of experiential solubility, the logarithm of the experimental solubility expressed as mole fraction. These two donors are shown together to allow a direct comparison under identical axis limits and a common color scale.

In Figure 7, GLY spans the central part of PC2 vs. PC1 and remains near moderate PC3 in PC3 vs. PC1, crossing regions where higher solubility is observed. TEG is more localized on the positive-PC1 side with elevated PC2 and moderate PC3, following compact regions where higher solubility is observed. This indicates complementary roles. GLY is suitable when broad compatibility is required, because it samples much of the mapped space while intersecting higher-solubility areas. TEG is appropriate when a focused placement within those areas is desired, as suggested by its concentrated footprint.

#### 2.5.3. Active Pharmaceutical Ingredients

**Figure 8:**
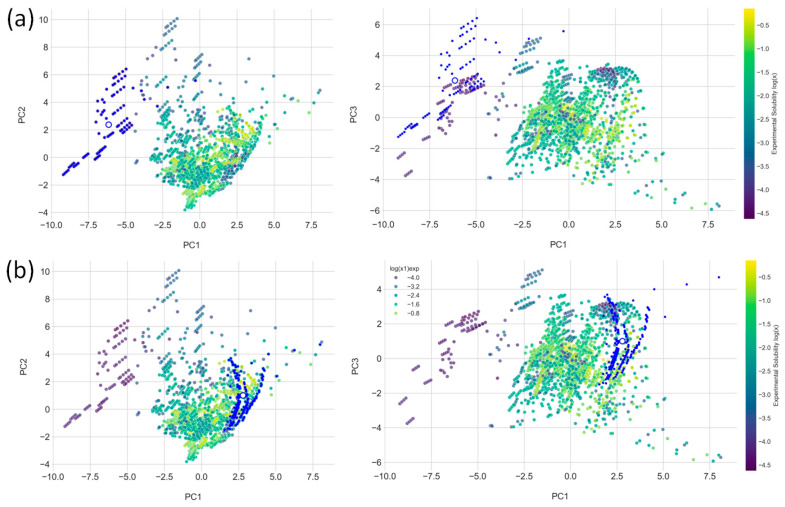
API-resolved projections of the DES solubility space for (**a**) Sulfasalazine and (**b**) Caffeine. Each API is shown as PC2 vs. PC1 and PC3 vs. PC1. Points are colored by the experimental solubility, log(x)exp, from the curated dataset (references [71,72,73,74,75,76,77,78,79,80]).

Two representative APIs were selected to clarify how positioning in PC space governs achievable solubility outcomes (Figure 8). Sulfasalazine serves as a demanding case with narrow formulation windows, whereas Caffeine exemplifies broad compatibility across the mapped space.

For Sulfasalazine, the API-resolved maps show a multi-cluster distribution dominated by low-solubility regions with only sparse, localized areas of improvement. Successful cases concentrate at moderate PC1 and within specific PC2 ranges, indicating that balanced global solvation combined with acceptable interaction matching is required.

Caffeine presents the opposite pattern. The API concentrates in the high-solubility regime and remains well represented among moderate performers, with only minimal presence in the lower-solubility clusters. The solubility overlay spans a wide portion of the map, indicating tolerance to PC1 variation and compatibility across a broad PC2 range. In practice, many DES compositions prove viable for this API, and only light optimization is generally required once basic compatibility is established.

For Sulfasalazine, the overlays cluster on the left side of the maps at strongly negative PC1 (about −8 to −5). In PC2 vs. PC1 they sit on a few discrete bands at positive PC2, and in PC3 vs. PC1 they occupy a narrow zone at PC3 around 2 to 3. These regions coincide mainly with parts of the background map where the solubility is lower. Only a few Sulfasalazine points approach the higher-solubility territory at PC1 greater than about 1 to 2. This pattern supports the view that Sulfasalazine has limited, localized windows of improvement.

Caffeine shows the opposite behavior. The overlays trace the right-hand arc at PC1 about 2 to 4 and are spread over PC2 from roughly −1 to 3 and PC3 from about 0 to 3. These areas align with regions of the background map where the solubility is higher, and Caffeine also remains present across adjacent moderate-solubility zones. In practice, this means many DES compositions are viable for Caffeine and only light optimization is typically needed once basic compatibility is identified.

### 2.6. Consideration of Temperature Effects

The present framework was constructed at a standardized temperature to isolate the dominant effects of DES and API chemistry. However, we recognize that temperature is a critical parameter for practical formulation. To evaluate its influence within our dataset, we analyzed a subset of systems for which solubility was measured at multiple temperatures. We observed that for a fixed DES-solute system, an increase in temperature primarily induces a positive shift along the PC1 (global solvation propensity) axis. This is mechanistically consistent, as higher temperature provides thermal energy to overcome the solute’s crystal lattice energy (a key driver of PC1) and reduces solvent viscosity, thereby enhancing mixing and dispersion interactions. In contrast, the coordinates on the PC2 (specific interaction complementarity) axis remained largely unchanged, as temperature has a lesser effect on the inherent hydrogen-bonding and polarity matching between solute and solvent. This preliminary analysis suggests that temperature acts as a modular scaling factor on the global solvation propensity, allowing for the prediction of temperature-dependent solubility behavior within the established PCA-clustering framework. A comprehensive, multi-temperature model represents a valuable and logical direction for future work to fully capture the thermodynamic landscape of DES solubilization.

## 3. Methods

### 3.1. Dataset and Response Variable

A curated dataset of 2010 solubility measurements for 21 active pharmaceutical ingredients (APIs) was compiled from our earlier studies [71,72,73,74,75,76,77,78,79,80]. Systems cover hydrogen-bond acceptors with and without added water, together with various polyol hydrogen-bond donors. The choice of hydrogen-bond acceptors (HBAs) and hydrogen-bond donors (HBDs) was guided by the need to represent both chemically diverse and pharmaceutically relevant deep eutectic solvent families. The HBAs included choline chloride, betaine, and menthol, covering three distinct physicochemical profiles: (i) Choline chloride is a prototypical ionic HBA, biocompatible and widely used in hydrophilic DESs; (ii) Betaine is a zwitterionic molecule offering intermediate polarity and enhanced hydrogen-bonding flexibility; (iii) Menthol—a hydrophobic, non-ionic HBA providing contrast in polarity and enabling assessment of amphiphilic DESs. HBDs, a homologous series of polyhydroxy alcohols (e.g., glycerol (GLY) and triethylene glycol (TEG) were selected. These compounds differ in chain length and hydrogen-bonding density, allowing systematic evaluation of how HBD functionality, polarity, and steric effects influence solvation efficiency. This design ensures coverage of both polar and amphiphilic DES domains, enabling quantitative comparison of solubility trends across chemically contrasting systems within a unified framework.

All determinations were carried out using the shake-flask method on the same instrumentation and with identical sample preparation, temperature control, and analytical readout, which ensures internal consistency and meaningful cross-system comparisons. Experimental solubility, log(x)exp, is reported as the logarithm of the measured mole-fraction solubility and is used consistently for map overlays and quantitative comparisons across figures. Solubility values can be found in the SM_dataset spreadsheet in the Supplementary Materials.

### 3.2. Molecular Descriptors

The COSMO-RS methodology [44,81,82,83] requires a faithful representation of conformational space; therefore, conformer analysis was conducted before evaluating thermodynamic properties. Using the default protocol, the COSMOconf [84] and TURBOMOLE [85] tandem was employed to produce representative structures for all solutes and solvents, in agreement with prior workflows [71,72,86]. For each molecule, no more than ten lowest-energy conformations were selected for gas- and condensed-phase representations, with solvent effects in the latter treated by the conductor-like screening model. The BP_TZVPD_FINE_24.ctd parameterization was applied to generate the required cosmo and energy files for COSMOtherm [87] at the RI-BP/TZVP//TZVPD-FINE level. Molecular descriptors were derived from COSMOtherm solubility outputs, which include interaction-energy analyses. Although the standard iterative solubility workflow is generally adequate, it often overpredicts complete miscibility for highly soluble solutes in DES systems [45,88,89,90]; in such cases, full solid–liquid equilibrium (SLE) calculations were performed. Fusion parameters for solid solutes—melting temperature (T_m_) and enthalpy of fusion (ΔH_fus_)—were compiled as averages of literature data [91]. The heat capacity change on fusion was taken as constant, and the entropy of fusion was approximated as ΔS_fus_ ≈ ΔH_fus_/T_m_, yielding ΔG_fus_ = ΔH_fus_ − TΔS_fus_; the values used are reported in the SM_dataset spreadsheet in Supplementary Materials. From the COSMO-RS output, five primary sources of solute descriptors were extracted: total intermolecular interaction energy (E_int,API_), its electrostatic misfit (E_misfit,API_), hydrogen-bonding (E_HB,API_), and van der Waals (E_vdW,API_) components, as well as the chemical potential (μ_API_). Corresponding sources of solvent descriptors (E_int,DES_, E_misfit,DES_, E_HB,DES_, E_vdW,DES_, μ_DES_) were obtained as mole-fraction-weighted sums over the DES constituents in the solute-free mixture. Relative (solute − DES) differences were included, as was the COSMO-RS predicted solubility, log(x_APICOSMO_). All descriptor values can be found in the SM_dataset spreadsheet in the Supplementary Materials, while Table 1 shows the detailed explanation of each descriptor.

### 3.3. Dimensionality Reduction and Stability Assessment

Principal Component Analysis (PCA) was applied to the matrix of 16 COSMO-RS–derived descriptors and fusion data to obtain an orthogonal, low-dimensional embedding suitable for interpretation and visualization. The analysis followed the standard PCA workflow on the descriptor matrix, producing scores for observations and loadings for descriptors. Four components were retained according to a predefined criterion that combined a target level of cumulative variance with evidence of loading stability under subsampling. Stability of component definitions was examined using a persistence analysis of loading vectors. PCA was recomputed on increasing random fractions of the dataset, and for each fraction the loading vectors were compared with the full-data solution to quantify convergence. Predefined interpretative thresholds were used when reading loadings: absolute loadings ≥0.35 were treated as strong contributions, values in the 0.24–0.35 range as moderate, and values below 0.24 as negligible. These thresholds guided the chemical labeling of components without relying on outcome magnitudes. The retained PCA scores were used subsequently as inputs to unsupervised clustering and for constructing the PC2 vs. PC1 and PC3 vs. PC1 maps reported in the figures, with identical axis limits across panels to enable direct comparison of subsets.

The interpretative thresholds for the PCA loadings (|0.35| for strong and |0.24| for moderate contributions) were determined based on the minimum significant loading value for a component. For a dataset with d variables (in this case, d = 16 descriptors), the minimum significant loading L_min_ for a given principal component can be estimated using the formula $L_{min}=5/\sqrt{d}$. This heuristic, derived from the work of Hair et al. [92], provides a cutoff to identify descriptors that contribute meaningfully to a component’s interpretation. Applying this to our descriptor set yields L_min_ ≈ 0.35. To ensure a more nuanced interpretation and avoid overlooking descriptors of moderate importance, we adopted a secondary, more liberal threshold of |0.24|, which corresponds to a loading that is approximately 70% of the minimum significant value. This two-tiered approach allows for a robust and chemically sensible interpretation of the principal components.

### 3.4. Unsupervised Clustering

To delineate recurrent solubility regimes, K-means clustering was performed in the four-dimensional PC space. The number of clusters was set to k = 4 based on an elbow analysis of the within-cluster sum of squares (inertia), which showed a pronounced inflection from roughly 22,500 at k = 2 to about 17,500 at k = 4, with diminishing returns beyond this point. This choice provided a balance between explanatory power and parsimony and yielded chemically meaningful partitions for interpretation.

### 3.5. Map Construction and Overlays

For visualization, data were projected as PC2 vs. PC1 and PC3 vs. PC1 planes using identical axis limits across panels. Points were colored by experimental solubility so that higher values mark regions of higher measured solubility. Subsets restricted to a single HBA, a single HBD, or a single API were plotted on the same axes to enable direct comparison with the global map.

## 4. Conclusions

This study establishes a robust, data-driven framework for classifying and predicting API solubility in deep eutectic solvents (DESs). By integrating COSMO-RS computations with multivariate statistics, we distilled 16 molecular descriptors into a chemically interpretable map defined by three principal axes: global solvation propensity (PC1), specific interaction complementarity (PC2), and bulk-medium efficiency (PC3). K-means clustering of this space revealed four distinct solubility regimes, providing a rational taxonomy for formulation design.

The key takeaways are threefold. First, the framework is diagnostic: a system’s cluster membership immediately identifies the primary solubility limitation, whether it is poor global solvation (Cluster 2), a lack of specific interactions (Cluster 3), or overriding crystalline stability (Cluster 0). Second, it is predictive and prescriptive: the path to high solubility (Cluster 1) is clearly guided by the principal components—optimizing PC2 through HBD/HBA complementarity is paramount, while PC1 and PC3 set the baseline and fine-tune performance. Finally, it is practical: the analysis yields direct design rules, identifying triethylene glycol (TEG) as a superior HBD for achieving high PC2 and underscoring the critical, almost universal, role of aqueous modification.

By translating complex solubility phenomena into a structured chemical space, this work provides a practical tool for replacing empirical screening with targeted DES design, accelerating the development of efficient pharmaceutical formulations.

## Figures and Tables

**Table 1:** Detailed explanations of the descriptors used in the study. The names of the descriptors are consistent with the terminology used in the SM_dataset spreadsheet in the Supplementary Materials.

Descriptor	Explanation
log(x_COSMO)	COSMO-RS-derived solubility collected as decadal logarithm of mole fraction
dμ	Relative value of chemical potentials (μ): $d\mu =\mu_{API}-\mu_{DES}$
dE_tot	Relative value of the total interaction energies (denoted in the output of COSMO-RS computations as “Total mean interaction energy in the mix (H_int)”): $dE\_tot=E_{API}^{tot}-E_{DES}^{tot}$
dE_Misfit	Relative value of the electrostatic contribution to intermolecular interaction energies (denoted in the output of COSMO-RS computations as “Misfit interaction energy in the mix (H_MF)”): $dE\_Misfit=E_{API}^{Misfit}-E_{DES}^{Misfit}$
dE_HB	Relative value of the hydrogen bonding contribution to intermolecular interaction energies (denoted in the output of COSMO-RS computations as “H-Bond interaction energy in the mix (H_HB)”: $dE\_HB=E_{API}^{HB}-E_{DES}^{HB}$
dE_vdW	Relative value of the non-bonding contribution to intermolecular interaction energies (denoted in the output of COSMO-RS computations as “VdW interaction energy in the mix (H_vdW)”: $dE\_vdW=E_{API}^{vdW}-E_{DES}^{vdW}$
μ_solvent	Values of chemical potential of solvent: $\mu \_solvent=\mu_{DES}=\sum_{i=1}^{N=3}x_{i}^{*}\cdot \mu_{i}\cdot$ computed as a weighted sum of component contributions, $x_{i}^{*}$ is the mole fraction of i-th component in solute solute-free solution.
E_tot_solvent	Values of the total interaction energies of DES $E\_tot\_solvent=E_{DES}^{tot}=\sum_{i=1}^{N=3}x_{i}^{*}\cdot E_{i}^{tot}\cdot$
E_Misfit_solvent	Values of the electrostatic interaction energies of DES $E\_Misfit\_solvent=E_{DES}^{Misfit}=\sum_{i=1}^{N=3}x_{i}^{*}\cdot E_{i}^{Misfit}\cdot$
E_HB_solvent	Values of the hydrogen bonding interaction energies of DES $E\_HB\_solvent=E_{DES}^{HB}=\sum_{i=1}^{N=3}x_{i}^{*}\cdot E_{i}^{HB}\cdot$
E_vdW_solvent	Values of non-bonding interaction energies of DES $E\_vdW\_solvent=E_{DES}^{vdW}=\sum_{i=1}^{N=3}x_{i}^{*}\cdot E_{i}^{vdW}\cdot$
μ1_sat	Values of chemical potential of the solutes: $\mu 1\_sat=\mu_{API}$
E1_tot_sat	The values of the total interaction energies of solute $E1\_tot\_sat=E_{API}^{tot}$
E1_Misfit_sat	Value of the electrostatic contribution to intermolecular interaction energies the solutes: $E1\_Misfit\_sat=E_{API}^{Misfit}$
E1_HB_sat	Values of the hydrogen bonding contribution to intermolecular interaction energies of the solutes $E1\_HB\_sat=E_{API}^{HB}$
E1_vdW_sat	Value of the non-bonding contribution to intermolecular interaction energies of the solutes $E1\_vdW\_sat=E_{API}^{vdW}$

## Data Availability

The original contributions presented in this study are included in the article/Supplementary Materials. Further inquiries can be directed to the corresponding author.

## Supplementary Materials

The following supporting information can be downloaded at: https://www.mdpi.com/article/10.3390/molecules30234563/s1, Supplementary Materials: SM_dataset spreadsheet.
